# The Foreign Journals: Pathology, Practical Medicine, and Therapeutics

**Published:** 1839-01

**Authors:** 


### PATHOLOGY, PRACTICAL MEDICINE, AND THERAPEUTICS.

### *On Simple Chronic Ulcer of the Stomach.* By M. CRUVEILHIER.

THE researches of M. Cruveilhier on the pathological anatomy of the stomach, lead him to conclude that there exists occasionally simple chronic ulceration of that organ, essentially different from cancerous ulceration of the stomach, but often presenting similar symptoms. There is indeed no pathognomic symptom which will enable us generally to distinguish the two diseases with precision, although there are circumstances which will guide our diagnosis. The natural termination of the two diseases differs essentially; as, whilst cancer of the stomach has an inevitably fatal tendency, simple chronic ulcer will often heal under a soothing treatment, and after a careful abstraction of all irritants. Yet this disease will sometimes terminate fatally by causing perforation of the stomach, or excessive hemorrhage; and even when the ulcer has healed, it will leave permanent ill consequences in some cases, producing a contraction of the stomach unfavorable to the passage of food, or attacking the coats of the arterial trunks which lie beneath the cicatrix.

1. Simple chronic ulcer of the stomach consists in a spontaneous loss of substance, generally circular, with sharp borders, dense and gray at the bottom, and of variable dimensions. There is rarely more than one, and this is situated in the small curvature or posterior part of the stomach. When it attacks the pylorus, it assumes the form of a zone. Its progress is slow, and as it extends in surface it increases in depth.

2. This kind of ulceration presents the same characters as cutaneous ulcers produced by a constitutional or local cause. It frequently resembles a syphilitic ulcer, but there is no ground for attributing to it a syphilitic origin.

3. Simple ulcer of the stomach may be distinguished from cancer by the absence of the hypertrophied and hardened base which accompanies scirrhous ulceration.

4. All the causes of gastritis are capable of producing simple ulcer of the stomach; but it is not uncommon to find this lesion in the bodies of persons who, during life, presented no symptom of it whatever. More generally, however, symptoms similar to those of scirrhus characterize it. Thus there is a failing or capricious appetite, insurmountable lowness of spirits, pain at the epigastrium increased during digestion. This pain frequently extends to the corresponding portion of the vertebral column, where it is felt with greater intensity than anteriorly. Emaciation, constipation, nausea, and vomiting of food, as well as vomiting of blood or a black matter, present a train of symptoms so similar to those of cancer, that it is only the experience of the effects of remedies which enables us to pronounce on the exact nature of the disease. It may, perhaps, be said that in cases of simple ulcer the patient is not so completely weighed down by the symptoms as in scirrhus.

5. If we examine the surface of the ulcer under water by the aid of a good magnifier, or even with the naked eye, we see a number of vascular orifices, some obliterated, others still open. It is from these orifices that the blood is poured out which sometimes produces alarming hæmatemesis. The black, sooty colour of the vomited matter arises from blood which has remained some time in the stomach, and has undergone the action of the gastric juice. When the ulceration attacks a vessel of considerable size, a quantity of blood may be poured into the stomach and bowels, the loss of which is sufficient to cause death. This termination is more frequent in simple ulcer than in cancer. Sometimes indeed the ulcer is completely cicatrized in every point except that which corresponds to a perforated vessel. In this case, the giving way of the coagulum may produce a fatal hemorrhage.

6. An absolute diagnosis between this disease and cancer is of the less consequence, because the treatment should be nearly the same. Repose of the stomach, as complete as the supply of the absolute wants of nature will allow, is essentially necessary. It is impossible to say beforehand what diet the ulcerated organ will bear; this must be determined in each case by careful experiment. Some will bear fish or white meat; others, veal or chicken-broth; whilst others will endure merely water containing sugar or gum in solution, or even simple water. The treatment should be commenced by enjoining on the patient complete abstinence even from liquids for twenty-four hours. If there is pain at the epigastrinm, leeches should be applied, and should be followed by a bath. The day after, a milk diet should be tried. A few spoonfuls of new milk taken occasionally will often suffice and agree well with the patient; if this is not the case, recourse must be had to gelatinous or farinaceous food, and the desires of the patient must be consulted. His natural instinct will often guide us to the discovery of the most proper aliment. Calcined magnesia is occasionally useful; opium rarely: sugar is in general injurious. Baths of gelatine are a very powerful auxiliary, and are much more useful, when they are continued during a space of two, three, or four hours. In this, as in many other chronic affections, it is important not to prolong the system of abstinence too far. Occasionally a change to more stimulating food, as game, will be of service. It is in the details of such a case that there is most room for the exercise of the sagacity of the practitioner. Quality and quantity of aliment; number and period of meals; temperature; period of exercise; excretions; all these points require the minute attention of the physician, and it is by attending to them, rather than by the action of medicines, that we may hope for the best results.

The cicatrices of the ulcers are all fibrous, very resistent, and consequently fragile. It is erroneous to state, that the losses of substance of mucous membranes are replaced by an accidental mucous tissue: a fibrous tissue not covered with a mucous layer replaces the portion of destroyed stomach. Recovery from ulceration, by no means renders the patient less exposed to perforation and hemorrhage.

The cicatrization of losses of substance of the stomach, similar to the same process occurring in the skin, is performed in two ways: 1, by the drawing together of the edge of the wound; 2, by the production of a fibrous tissue. Inconsiderable losses of substance are cured exclusively by the first method, and then the cicatrix of the stomach is represented by a linear stroke or by a small depression, with circular puckering and radiated folds. More considerable losses of substance leave a circular depression, as if made by a punch, which has a fibrous bottom, limited by a border of mucous membrane, more or less projecting.

Perforations may happen during the period of improvement of the ulceration, when the necessary adhesions are not established, and then it may be the result, 1st, of a new ulceration, which may occur on the very bottom of the cicatrization, or upon a point of its circumference; 2dly, from a default of extensibility, from the fragility of the cicatrix, which is broken in consequence of distension of the stomach, either by gas or by aliments, or from violent vomiting.

Hemorrhage may take place: 1st, during the period of increase of the ulcer; it is then the result of erosion of the arterial walls; 2dly, after the cicatrization, and then sometimes it is produced by the fall of an obstructing clot; sometimes it is in consequence of an ulceration which invades a portion of the cicatrix, or a process of erosion limited to a vessel.

Death is the certain and inevitable consequence of perforation. The hemorrhage may be either rapidly fatal, or the patient may sink from a succession of attacks. The blood is generally passed by vomiting, it is often passed both by vomiting and by stools. In some cases altogether by the stools which are like ink: in these cases it is probable that the blood is furnished in small quantities, and is in some measure digested.

Those patients who have been cured of this disease are prone to a return from the slightest causes. M. Cruveilhier has seen it reproduced in the same patient three times, at intervals of from two to four years. The knowledge of a patient having recovered from such an attack, should indicate great caution in the employment of irritating medicines. Two cases are given: we select the last, because it clearly points out the importance of bearing in mind this important observation.

A female, aged sixty, entered the hospital on the 23d of September, 1834, for a prolapsus of the rectum. The attention being directed to this point only, the remedies for such an affection only were ordered. Some days after, upon the patient complaining of a bad taste and of habitual constipation, she was ordered twenty-four grains of ipecacuanha with relief. On the following day, she complained that her food produced a very painful sensation in the epigastric region, and it was at this period she gave the history of her case. She had been seized eighteen months before with vomiting, pains in the stomach, and fever. The vomiting was produced by the ingestion of food, and occurred from time to time, accompanied with epigastric pains; these were so violent as to give the sensation of an animal in the stomach. Pressure upon the epigastric region caused a very acute pain, but no tumour could be discovered. Cataplasms and milk diet were ordered. On the 4th of October, she was suddenly seized with an acute abdominal pain, agitation, and anguish. The following morning there were a distressed and discoloured countenance, miserable pulse, cold perspiration, acute sensibility of the abdomen; more especially of the epigastrium and the left hypochondrium, nausea and hiccup. M. C. diagnosticated a perforation of the stomach: fifteen leeches and cataplasms were applied, but the patient died during the night.

*Dissection.* The intestines were injected and covered with a yellow, false membrane, mixed with bile and alimentary matter. The pelvis contained several ounces of a turbid serum, in which were several seeds and skins of grapes. The intestines were united together. The stomach presented in front, very near the great curvature, not far from the pylorus, a round perforation, about two lines in diameter. The stomach when opened presented, on a level with this perforation, a recent ulceration of an oval form. Independently of this recent ulceration there existed upon the posterior wall, on a level with the pancreas, a cicatrix formed by this organ, but covered by a layer of smooth fibrous tissue. The mucous membrane formed a circular pad, but it was in a manner continuous with the tissue of the cicatrix. M. C. believes that the irritating nature of the emetic caused the return of the disease.

Many other cases are related, illustrating the termination of this disease. Four cases are detailed in which the stomach was perforated by the ulceration; its contents escaped into the cavity of the abdomen and death ensued in a few hours. Two cases are given in which the disease terminated by fatal hæmatemesis; in one, the coronary artery, in the other the splenic, was the vessel attacked. In both cases the ulcers had cicatrized, except at the points corresponding to the vessels from which the hemorrhage proceeded, and to a small extent around these points. Three cases are given in which examination after death showed the existence of cicatrized ulcers in the stomach of persons, who at a former period had suffered from symptoms of gastritis. Such was the case of Professor Beclard. In one case, death was produced by excessive hemorrhage from the stomach, but on inspection no open vessel was found, and the extreme development of the superficial veins supported the idea that the hemorrhage had taken place by exhalation.

[We had marked for extract, as the best possible commentary on this paper—or rather as its complement—the admirable essay on the same subject, by Mr. Langston Parker, published in the October Number of the Medico-Chirurgical Review: we regret, however, that the pressure of other valuable matter, less accessible to the English reader, obliges us to content ourselves with referring the reader to it. It will repay the most careful perusal.]

*Revue Médicale. February, March, and July,* 1838.

---

*On the Miliary or Sweating Fever* (*Suette Miliaire*), *Epidemic at Vesoul (France) in the months of March and April,* 1837. By F. Pratbernon.

M. Pratbernon considers this disease, which he has described as occurring epidemically at Vesoul, as a specific affection characterized by very marked symptoms, and closely agreeing in its characters with the epidemics which have at different times ravaged Europe, particularly in the fifteenth and sixteenth centuries, and which were named from their most prominent symptom the sweating sickness. He says that this frightful disease is by no means of rare occurrence in that portion of the department of the Upper Soane which extends from the foot of the Vosges to the capital, Vesoul; he has met with many cases during the last five years. It generally occurred at the end of the winter, killing most of those whom it attacked with the rapidity of the plague. Between the 4th and the 27th of March, 1837, fifty adults were attacked out of a population of 6,000; twenty of these died, some in less than a day. By the common people, the disease was called *the purples*. It generally commenced with a feeling of languor and general feverishness, accompanied sometimes with catarrhal symptoms: after a few days the fever became violent; the pulse was quick, small, and soft; the countenance anxious; the skin hot and red at first, and soon covered with profuse sweating, the perspiration rising like smoke or vapour from all parts of the body, and penetrating the bed clothes; it possessed a peculiar sour smell at first, and afterwards became very fætid, its odour being compared to that of rotten straw. Generally about the third day an eruption of small, red papillæ broke out, which became vesicular: the vesicles contained at first a serous fluid, which afterwards turned purulent; they lastly dried up and formed scales which were detached with the cuticle during convalescence. The sweating became less abundant after the appearance of the eruption. Sometimes the eruption was partial, and irregular in its appearance and continuance. The eruptive stage was that of the greatest danger. There was generally a feeling of oppression at the chest and pit of the stomach, with palpitation of the heart, and a great dread and presentiment of death. M. Pratbernon remarked that the symptoms had a deceitful mildness, till within a short time of death; they then became suddenly much aggravated, the breathing was greatly oppressed, the trachea seemed obstructed with frothy mucus, and the patient died as if asphyxiated.

Putrefaction took place so rapidly after death even in the coldest weather, that it was found mostly impossible to make any examination of the body. M. Pratbernon relates five cases, three of which were fatal, and in two of which a careful post-mortem investigation was made soon after death, but no peculiar lesions were met with; the lungs were gorged with blood, and the mucous membrane of the trachea and bronchi was deeply injected. The vessels of the brain were also gorged, and there was a considerable effusion of serum between the membranes of the brain, and into the spinal canal. The blood was in a fluid, dissolved state.

With respect to the *causes* and *treatment* of this disease, neither of them seem much understood; the latter was varied according to the circumstances of the case: the most successful plan seemed to be to keep the patient moderately warm, and give mild acidulated drinks; an emetic appeared sometimes to do good in the early stages, and sulphate of quinine produced beneficial effects towards the termination of the case. Much obscurity hangs over the source or mode of origin of this, as well as many other diseases. It seemed to be epidemic, or rather endemic, for it was confined to certain restricted localities. By some it was considered as contagious; though this opinion was contrary to the evidence of facts, for the disease was frequently confined to one individual in a family. On the other hand, it could not be traced to the influence of marsh miasmata, for it often appeared in dry situations, as paved towns, while low, swampy villages escaped. The source of the disease seemed, however, very confined in its range, and M. Pratbernon thought it was owing to some local or domestic causes. He suspected that it might arise from putrid exhalations engendered by the common people living together in crowded situations, and being in the custom of sleeping between two beds made with soiled greasy feathers, procured from numerous wild-fowl with which the country abounds. This sweating fever generally attacked people in the prime of life, avoiding children and old persons, and it is stated that three times more females than males fell victims to it; newly-confined women seemed particularly obnoxious to its influence.

*Revue Médicale française et étrangère. August*, 1838.

---

### *On the Exhibition of Hydriodate of Potash in secondary Syphilis, Scrofula, &c.* By Dr. VON HASELBERG.

[THE following cases are related as corroborative of Wallace's views of the efficacy of hydriodate of potash in secondary syphilis; but we are inclined to think that sufficient time was not allowed in most of the cases to warrant the patient being considered secure against a relapse.]

CASE I. A woman, aged sixty-three, was affected with small itching ulcers on the vagina and neighbouring parts, with numerous copper-coloured patches on various parts of the body, and with difficulty of deglutition and pain and swelling in the throat. Corrosive sublimate was exhibited, and between the 29th August and 3d October she took sixteen grains of the salt. Under its use the ulcers on the genital organs and the maculæ on the body disappeared; but the redness and swelling of the throat increased, and ulcers formed on the tonsils. Recourse was now had to the hydriodate of potash, and a table-spoonful of a solution, containing eight grains to four ounces of water, gradually increased to double the strength, was exhibited three or four times daily. Improvement was manifest before half a drachm of the salt had been taken, and eighty grains were sufficient to procure the cicatrization of the ulcers. Its use was now discontinued, but in a few weeks the pain in the throat returned: its exhibition was therefore again resumed, and continued with slight interruptions, which were always followed by an increase of the disease of the throat, for about two and a half months, when all trace of the disease in the throat was removed. The patient died three months afterwards from apoplexy, without a relapse having taken place.

Case ii. In 1816 Mr. C. contracted a gonorrhœa, for which he was salivated; and in 1819 he was affected with syphilis, and again took mercury. In 1834 he applied to Dr. Haselberg on account of a painful periostosis on the right clavicle, accompanied by pains in the shoulder-joints and in the neck and head. Dzondi's mercurial treatment was then commenced; but the extreme weakness of the patient, and the early appearance of salivation, prevented it from being fully carried through. The tumour on the clavicle was greatly diminished, but most agonizing pains continued to affect the whole osseous system. This state continued in spite of the use of a great variety of remedies for about two years, the patient steadfastly refusing to submit again to mercurial treatment. In the autumn of 1836, the pains in the skull became, if possible, still more severe, and the periosteum of the frontal bone began to press upon the eyeball. The exhibition of hydriodate of potash was now resolved upon, and its use was accordingly commenced on the 9th of November. On the 3d of December the patient expressed himself much relieved, and the amendment continued till the 20th, when the use of the hydriodate was interrupted; one ounce and a half having been taken. Its exhibition was recommenced in January, as the pains returned with as great intensity as ever; and on the 4th of March three ounces additional had been taken, with almost complete relief. It was again interrupted, and in fourteen days the pains were as violent as ever. Calomel and opium, in minute doses, were now administered, but almost immediately stopped, as salivation ensued. On the 4th of May the hydriodate was again given; and on the 20th of June three ounces additional had been consumed, with great benefit to the patient. The use of the salt was now desisted from, and, on the 27th of September, the patient still continued in excellent health, and free from all pain.

Case iii. On the 10th October, 1835, a gentleman became aware of a small chancre on the prepuce, which at first was treated on the anti-mercurial plan; but calomel was afterwards exhibited till the gums were slightly touched. On the 4th November the health was considered as reestablished; but on the 16th December he complained of redness and pain in the tonsils, symptoms which were speedily followed by ulceration. The patient was treated by mercury between the 22d December and 21st January, and the symptoms were removed; but on the 15th of February the ulcers reappeared upon the tonsils. The red precipitate was now exhibited, and on the 17th of March the ulcers had cicatrized; twenty-eight grains of the oxide having been taken. The patient now continued in good health till the 3d of August, 1837, (sixteen months after his apparent cure,) when an ulcer appeared upon the right tonsil. The hydriodate of potash was exhibited upon the 5th of August, and its use continued till the 20th of September; when the patient was considered not only as completely restored, but as safe against relapse.

[Several other cases are detailed, in which the hydriodate acted beneficially for a time at least; but, as the history of the patient is not continued beyond the period at which the exhibition of the salt is stopped, it is impossible to say whether the benefit proved lasting or merely temporary: we think it needless, therefore, to give them here.]

*Medicinische Zeitung.* 1837. Nos. 48 and 49.

*Two Cases of Facial Paralysis.* By Dr. C. J. HEIDLER, of Marienbad.

[THE two following cases are related as evidence of the efficacy of emetics in removing paralysis of the facial nerve.]

A man, æt. 44, of rather feeble constitution, complained of a feeling of fulness in the head after a morning of rather severe study. In the afternoon, during an excursion to the country, he exposed himself to cold, and on his return home became aware of a slight difficulty of rounding the mouth in spitting. This symptom gradually increased till the case assumed the aspect of a mild form of paralysis of one side of the face. There was still slight congestion of the brain, and the whole vascular system was a little excited. The tongue was affected only in a slight degree. The diagnosis was—a congested, or perhaps an inflammatory state of the root or trunk of the facial nerve. Venesection of twelve ounces, a blister to the nape of the neck, cold applications to the head, and an emetic. The only immediate consequence of these remedies was the partial relief of the congestion of the head. The patient passed a good night; but on the second day the paralysis seemed to have increased rather than to have diminished: leeches were, in consequence, applied behind the ears and to the nape of the neck, and sinapisms to the feet; the cold applications to the head were continued. In the afternoon a blister was applied behind the ear; the back and the calves of the leg were dry-cupped, and a drastic purgative prescribed; but these measures failed in producing any apparent diminution of the paralysis. On the afternoon of the third day, another venesection of ten ounces was made, in consequence of a return of the symptoms of cerebral congestion; ice was kept applied to the head, and in the evening an emetic was administered. This was followed in some hours by a sensible diminution of the paralysis and the disappearance of the cerebral fulness. On the fourth day warm cataplasms were applied to the affected cheek; but cold applications to the head were repeatedly substituted, when the symptoms of congestion threatened to appear. On the fifth day there was again slight congestion, for which leeches were applied, and an emetic given in the evening, but without in any degree removing the paralysis. On the sixth and seventh days the warm cataplasms were occasionally applied, and another emetic was administered in the forenoon of the latter day, which produced copious vomiting. In the evening the patient regained some power over the affected muscles, and during the two following days it continued to increase; the warm cataplasms being occasionally applied. On the eleventh day another emetic was ordered, which produced still further diminution of the paralysis, which eight days sufficed to remove entirely.

CASE II. A woman, æt. 40, of weak constitution, was affected with violent inflammation of the left ear, which apparently extended to the brain and corresponding facial nerve, leaving, after its removal, a total paralysis of the muscles of this side of the face. Four weeks after the inflammation had ceased, all the branches of the facial nerve, but particularly its trunk immediately after its exit from the cranium, still showed considerable sensibility on pressure. The brain was affected in a slight degree, and the whole of the left side of the head felt tight and uncomfortable. The commissure of the mouth and the eyeball were nearly immoveable, and considerable difficulty was experienced in moving the eyelids, which during sleep remained half open. The intellect was slightly affected, and a feeling of numbness in the hand and foot of the affected side gave rise to suspicions of threatening apoplexy. Such was the state of the patient in the sixth week after removal of the inflammation, notwithstanding the energetic employment of counter-irritation and other remedies. A slight indigestion was at this period the cause of an emetic being administered, which had the effect of producing a sensible diminution of the paralysis. The patient made no further advance towards recovery during the next eight days: a second emetic was then exhibited, which was followed by a similar diminution of the symptoms. Seven emetics were thus successively given at longer or shorter intervals, with the final result of removing completely the paralytic affection.

*Rust's Magazin.* Band xlix. Heft 2.

### *Accidental Development of a Canal filled with Serum in the centre of the Spinal Cord.* By M. Monat.

The following is the history of a curious case in which a multilocular canal filled with a limpid serum was discovered in the spinal cord, communicating with the fourth ventricle, by means of the *calamus scriptorius.*

A man, aged thirty-four, experienced, in October 1835, a pain in the inferior and posterior part of the neck, analogous to rheumatism; it afterwards left the cervical region and attacked the dorsal, producing cramps of the inferior extremities, with diminution of motion and increase of sensibility. Some time after, atony of the rectum and bladder was manifested, and after some months a complete paraplegia with œdema of the inferior extremities, sloughs upon the sacrum, &c. Nothing abnormal was observed in the intellectual manifestations, in the organs of the senses, nor in the parts situated above the umbilicus. There was great emaciation.

Such was the state of the patient upon entering the Hôtel Dieu on the 21st of September, 1836. It was supposed to be a case of ramollissement of the spinal cord. On the 2d of October, the respiration became embarrassed, and on the 5th he expired in a comatose state. The spinal cord was generally softened, especially the centre of the superior part which was almost fluid. On a level with the fifth cervical vertebra, in the centre of the cord, there was a cavity, filled with a sanguinolent black matter, mixed with the softened medullary pulp. An incision being made a little below, upon the median line, a transparent serous fluid flowed out, which occupied a sort of canal dug out of the thickness of the cord. It was prolonged as far as the eighth dorsal vertebra, and was interiorly lined by a species of serous tissue. The canal communicated with the fourth ventricle by means of the calamus scriptorius, and was divided into several cells by means of medullary partitions corresponding to each pair of nerves.

M. Monat regards this canal as accidental and not congenital.

*Archives Génerales de Médecine. March,* 1838.

---

### *On the Resistance offered by the Peritoneum to Perforation of the Intestine.* By Professor Sebastian.

An appearance illustrating this resistance in a remarkable manner presented itself in the small intestine of a youth who died of phthisis. There were numerous ulcers of different sizes in the mucous lining of the bowels, and at the same time several small tubercles in the cellular tissue between the muscular and serous coats. At one point a dependant sac formed by the peritoneal coat, and partly filled with bilious pus, was observed. Its interior communicated with the cavity of the intestine, and the muscular and mucous membranes of the latter were destroyed by ulceration to about an inch in extent round the orifice of the sac. This last had evidently been formed by a process of elongation, caused by the pressure of the intestinal contents after the destruction of the two other coats of the bowel. The whole was an accurate imitation of the state of the arterial membranes in Scarpa's aneurism. I am not aware whether in cases of perforated intestine the peritoneum usually yields in the manner described before the rupture. No mention is made of such a fact by authors who have given us cases of perforation; but the peritoneum which had been distended before might have contracted after the rupture.

[Notwithstanding the Professor's' suggestion, we still are of opinion that this elongation of the peritoneum must be, at the least, an exceedingly rare phenomenon. Admitting that contraction might take place after the perforation, there must still remain in the dead subject a loose portion of peritoneum corresponding to the pouch, which, had it existed in any of his cases, could not have escaped the vigilant eye of such an observer as Louis; now no such appearance is described by him in his cases of perforated ileum.]

*Tijdsch. voor Natuurl. Geschiedenis.* 3 *Deel.*

### *On Disengagement of a Gaseous Fluid in the Circulation, as a cause of Sudden Death.* By Dr. OLLIVIER.

AFTER noticing the common causes of sudden death, such as cerebral and spinal apoplexy, pulmonary apoplexy, and emphysema, rupture of the heart or of the large vessels, Dr. Ollivier asserts that many cases occur in which an examination after death demonstrates no organic lesion, and consequently that the cause of death is by no means apparent or satisfactorily accounted for. The author's object in the present paper is to prove the possibility of a spontaneous development in the blood during life, of a gaseous fluid, which produces instantaneous death by its accumulation in the right cavities of the heart; either as an obstacle purely mechanical to the circulation, if the gas is analogous to atmospheric air; or as a deleterious agent, if, as may be supposed, it is carbonic acid gas which is disengaged. The following is a case which is supposed to support this view.

A young woman died suddenly, at the moment she was endeavouring to get up. Upon examination nothing could be found to account for death, but a considerable dilatation of the right cavities of the heart by a gaseous fluid. She had been suffering for some time from debility which was sensibly increased, without any apparent cause upon the day of her death.

*Revue Médicale. February,* 1838.

---

### *Case of Congenital Transposition of the Viscera.* By Dr. TONELLI, of Rome.

A MALE infant, born at the full time, and in other respects properly developed, presented the following appearances when seen by the writer, on the third day from the birth. A tumour of oblong shape, resembling a moderate-sized cucumber, was attached to the umbilicus by a peduncle which spread out into a funnel-shaped base, about nine inches in circumference. The tumour itself extended longitudinally from the middle of the sternum half-way down the thighs. [Its longitudinal measurement is not more particularly stated.] It projected in front of the navel five or six inches. Its transverse diameter, opposite the navel, was about six fingers' breadth. The lower portion of its investing membrane was pellucid, yellowish, and evidently contained a fluid. The upper portion resembled the common integuments, and had a soft elastic feel. The navel-string adhered to the right side of the tumour for the space of about four inches. The infant had properly voided its urine and meconium, and had begun to take the breast. It died shortly after this inspection. On the lower part of the cyst being punctured, there flowed out about six ounces of yellowish serum. When the upper part was laid open, all the viscera were found within, arranged in a relative position analogous to that which they would have naturally occupied within the abdomen, with the exception only of the kidneys, bladder, and rectum. On tracing the cardiac extremity of the stomach, it was found to bend upwards, and enter the umbilical opening by the side of the umbilical vessels. In like manner the extremity of the colon was seen to enter the abdomen. The cyst was now removed by cutting through the peduncle, the orifice of which measured two inches and a half in diameter. The abdominal cavity was found rather smaller, and the tendinous attachments of the diaphragm, posteriorly, somewhat lower down than natural. The kidneys and urinary bladder were found in their natural positions, and the stomach and colon were found continuous with the œsophagus and rectum respectively, as was to be expected from the regular performance of the functions of those organs. Dr. Tonelli considers the present case as one not hitherto described. He refers to the case of thoracic hernia recorded by Mr. Morgan, of the Bristol Infirmary, and to the congenital transposition of the viscera in an adult observed at Calcutta, by Mr. Hardy, (see both articles in Medical Gazette, vol. xii. pp. 79 and 673,) but justly remarks that these cases bear no analogy with the present one.

*Annali Universali di Medicina.* Vol. lxxxii. *Aprile, Maggio, Giugno,* 1837.

